# Sense of Coherence in Association with Stress Experience and Health in Adolescents

**DOI:** 10.3390/ijerph17093003

**Published:** 2020-04-26

**Authors:** Unni Karin Moksnes, Geir Arild Espnes

**Affiliations:** 1 Department of Public Health and Nursing, NTNU Center for Health Promotion Research, Norwegian University of Science and Technology, 7030 Trondheim, Norway; 2 Faculty of Nursing and Health Sciences, Nord University, 8026 Bodø, Norway

**Keywords:** subjective health complaints, self-rated health, mental health, stress, sense of coherence, salutogenesis, moderator

## Abstract

This study investigated the associations between sex, age, socio-economic status, stress, sense of coherence (SOC), and health (mental wellbeing, depressive symptoms, self-rated health, and subjective health complaints) in Norwegian adolescents aged 13–19 years. Furthermore, the study investigated the potential protective or compensatory role from SOC on the association between stress and health. *Methods:* The study was based on a cross-sectional sample of 1233 adolescents. Data were analyzed with descriptive, comparative, and multiple linear regression analyses. *Results*: Girls reported significantly higher scores on depressive symptoms and subjective health complaints than boys. Stress was significantly and positively associated with depressive symptoms. SOC associated significantly with all outcome variables; and especially with mental wellbeing and depressive symptoms. Significant interaction effects of sex in combination with stress and SOC were found in association with depression and mental wellbeing. Associations were strongest for girls. *Conclusion:* The findings provided support for the significant role of SOC as a coping resource, especially in relation to adolescents’ mental health; weaker associations were found with subjective health complains and self-rated health. The findings also mainly supported a compensatory role of SOC on the association between stress and health during adolescence.

## 1. Introduction

A fair opportunity for every young person to reach their full health potential is a democratic goal for most societies, regardless of demographic, social, economic, educational, and cultural factors [1,2]. Hence, in order to promote positive development in adolescents it is important to investigate how adolescents evaluate their health, and what factors have the greatest impact on their health, as assessed through self-reports. This was also interesting in reference to the fact that young people especially during this period of life experience changes and transitions, which might influence their health and well-being throughout the life course [3,4].

In general, in Norwegian and most other Western societies, children and adolescents growing up today are characterized by good health and a high quality of life. However, self-reported mental health problems have increased in recent years, both globally and nationally and account for a large proportion of negative health outcomes in young people, in all societies [5,6,7]. In Norway, it is estimated that approximately one in five adolescents have mental health problems affecting their daily life and seven percent have symptoms that meet the requirements for a psychiatric diagnosis [8]. Mental health problems seem to be especially evident in girls, where the proportion of girls aged 15–20 years who are given a psychiatric diagnosis (most common problems are depression, anxiety, eating disorders, and behavioral disorders), has increased from five to seven percent per year, from 2011 to 2016. [8]. 

Adolescents typically have low rates of serious medical illnesses, but studies show an increase in reports of subjective health complains (SHC), especially among girls, during the adolescent years [9,10,11,12]. These complaints refer to mental and physical ‘unexplained symptoms’, often related to stress experience [9,10,11,12]. A well-used indicator to assess the overall perception of health status, is to ask people to self-rate their health (SRH) [13,14]. Previous studies suggest that adolescents’ perception of health seem to be relatively stable during the adolescent years [13,14,15,16]. However, sex differences in SRH are often reported to increase with age, where especially girls seem to report more negative evaluations of SRH than boys [9,10,11,17,18,19]. There is evidence to show that this health deterioration, along with an increase in SHC, relates to a broad spectrum of medical, physical, psychological, and psychosocial factors, where an increased experience of multiple independent and cumulative stressors is recognized as one important factor [10,17,20,21]. Research shows that stress levels increase from preadolescence to adolescence, where girls report higher stressor load and seem to be more vulnerable to the negative psychological effects of stress than boys [4,20]. In order to promote positive functioning, health, and wellbeing in the adolescent population, it is important to gain a better understanding of how stress relates to adolescents’ overall experience of health, as well as investigating the role of potential protective factors in this context. The concept of sense of coherence (SOC) is central in the exploration of what coping resources are crucial for the individual’s capacity to cope with stressors in daily life and create health (salutogenesis) as a complementary approach to the traditional focus on risks for disease (pathogenesis) [22,23]. SOC is described as a personal coping resource and life orientation, which is recognized as the ability to perceive life as comprehensible, manageable, and meaningful, and the perception of having resources needed to cope with normative and non-normative stressors in daily life [22,23].

SOC is a central resource for the protection and promotion of health [24]. A strong SOC is associated with a positive mental health and subjective well-being and a lower severity of symptoms of anxiety and depression [22,24,25,26]. Through the last years, a discussion has evolved regarding the weak associations between SOC and physical health [27]. This has been explained by the fact that SOC mainly comprises the individual’s mental, social, and spiritual resources for coping with life challenges [24]. Studies in adolescent samples have, however, shown positive associations between SOC and perceived positive health [28,29,30], and negative associations between SOC and SHC [17,31,32]. Where adolescents have been examined for ‘normal’ life stressors, such as academic, school, or peer pressure as well as family conflicts, it has been shown that those with stronger SOC report lower stress levels [26,32,33,34]. 

It is unclear whether SOC has a compensatory or protective role on the association between perceived stress and health. A compensatory model proposes that SOC operates as a resource, irrespective of stress levels (compensation), while a protective model claims that SOC is activated in the face of adversity (buffer effect). In adult samples, SOC seems to have both a protective and compensatory role in association with different health outcomes [24,35]. Studies conducted in adolescents focusing on daily life stressors have shown that SOC has a weak-to-moderate stress protective role in relation to SHC [21,31,36]. In studies based on Norwegian adolescent samples, support for a stress compensatory role of SOC has mainly been found in relation to both SHC [17], life satisfaction [37], and symptoms of anxiety and depression [26]. These studies have similarities with the present study by investing the role of stress and SOC in relation to mental and physical symptoms. However, the present study extends these studies by investigating the health outcomes more broadly, including subjective-, physical-, and mental health, as well as investigating the potential moderating role of sex and SOC on the association between stress and health in a sample of Norwegian adolescents age 13–19 years in rural areas in mid-Norway. The present study also included socio-economic status that are relevant to investigate in relation to adolescents’ health and wellbeing [1,2].

The aims of the study were to investigate in adolescents:

1. Sex differences in self-reported health (SHC, SRH, mental wellbeing, and symptoms of depression);
2. The relation between stress, SOC, and health; and potential sex differences in these associations;
3. The potential protective or compensatory role of SOC on the association between stress and health.

## 2. Method

### 2.1. Participants

This cross-sectional study was based on data from adolescents in public lower- and upper-secondary schools, in five municipalities from inland and coastal rural areas in the county of Trøndelag, located in Central Norway. The schools offer vocational and academic study tracks that are representative of Norwegian upper secondary schools. In the data collection from 2016, 1906 students were invited to participate in the study, with N = 1282 responding on a questionnaire (a response rate of 67%). Non-responses were caused by students not being at school at the time of data collection, non-willingness to participate or because some classes did not have the chance to participate as the teachers could not administer the questionnaire. No detailed information was available on non-responders. Adolescents <13 or >19 years (n = 49) were excluded, resulting in n = 1233 (64%) being included in the study sample (Table 1).

### 2.2. Procedure

Data collection was approved by the Regional Committee for Medical Research Ethics (approval number 2016/1165). Prior to data collection, a written information letter was sent to all students and to parents of those ≤15 years, underscoring that participation was voluntary and anonymous, that participants were free to withdraw from the study, and that the collected information was treated with confidentiality. According to research ethical guidelines, written consent was required from adolescents and their parents when adolescents were ≤15 years. Adolescents ≥16 years gave consent by answering the questionnaire. Questionnaire administration was completed with help from teachers in whole class groups during one regular school session (of the teachers’ choice) of 45 min, in 2016.

### 2.3. Measures

Self-rated health (SRH) was assessed by one item, ‘‘How is your health now?’’ The response options were: 1–‘bad’, 2–‘not so good’, 3–‘good’, 4–‘very good’, and 5–‘extremely good’. Assessment of health using one item was previously found to be satisfactory for use in other studies on adolescents’ health [13,14].

Subjective health complaints (SHC) was measured by 12 items comprising physical symptoms (e.g., stomachache, headache, pain in the back/arms/legs, and cold) and mental symptoms (e.g., bad mood, felt lonely, nervous, sad, or irritable). Participants responded on a four-point scale ranging from 1–‘not bothered’ to 4–‘very much bothered’, where higher sum scores indicated higher symptom load. Cronbach’s α for the instrument was 0.86.

Sense of coherence (SOC) was assessed with the 13-item Orientation to Life Questionnaire consisting of 13-items rated on a seven-point scale; higher sum scores indicated stronger SOC. The questionnaire has been extensively validated and used cross-culturally, both in adult and adolescent samples [38,39]. In the present study, Cronbach’s α was 0.82.

Adolescent stress was measured by use of the Norwegian 30-item version of the Adolescent Stress Questionnaire (ASQ-N). Each item was rated on a five-point Likert scale ranging from 1–‘not at all stressful’ or ‘irrelevant to me’ to 5–‘very stressful’, where a higher sum score indicated higher stress level. The scale was validated for use in Norwegian adolescents [40] and adolescents in other European countries [41,42,43]. Cronbach’s α for the instrument in the present study was 0.94.

Mental well-being (MWB) was assessed with the 14-item version of Warwick–Edinburgh Mental Well-Being Scale (WEMWBS) [44]. The respondents were asked how they had felt about seven positively worded statements over the past two weeks. The values ranged from 1–‘None of the time’ to 5–‘All of the time’, where higher sum scores indicated higher levels of mental well-being (range 14–70). The WEMWBS was validated in the general population [44,45], clinical samples [46], and in adolescents [47,48,49]. Cronbach’s α for the scale in the present study was 0.91.

Symptoms of depression was measured using a non-clinical depression scale appropriate for measuring non-clinical depressive attributes [3]. The scale consisted of a 15-item questionnaire measuring respondents’ levels of current depressive moods. Item choice was informed by reference to commonly experienced depressive features outlined in the Diagnostic and Statistical Manual–Fourth Edition [50], and to the Zung Self Rating Depression Scale [51]. The items were rated on a 5-point Likert scale ranging from 1–‘never’ to 5–‘always’, where higher scores indicated a higher symptom load. The scale was used in previous studies in the adolescent population [26,40] Cronbach’s α for the instrument in the present study was 0.94.

Socioeconomic status (SES) was measured in terms of mother’s and father’s education, employment status, and adolescents’ perception of their family’s economic situation. Mother’s and father’s education were assessed separately using one item: “‘What is your parents’ highest education?”; 1–‘Primary and lower secondary school’, 2–‘Upper secondary school’, 3–‘University up to 4 years’, 4–‘University, more than 4 years’, 5–‘Don’t know’. Mother’s and father’s employment status was assessed separately with the item “‘What is your parents’ employment status?”; 1–‘stay at home’, 2–‘unemployed’, 3–‘part time job’, 4–‘full time job’, 5–‘other’. Adolescents’ perception of family economy was assessed by one item: “How has the family economy been during the last two years?”; 1–‘We have had bad economy the whole time’, to 5–‘We have had good economy the whole time’.

### 2.4. Statistical Analyses

Statistical analyses were conducted using SPSS, version 22.0 BM SPSS, Armonk, NY, USA. Descriptive statistics included frequencies, means, and standard deviations. T-tests were calculated to test sex mean differences on the scales in the study. To evaluate the strength of the sex mean differences, effect sizes were calculated following Cohen’s [52] guidelines for small (0.20), medium (0.50), and large (0.80+) effect sizes. Bivariate correlations between the continuous variables of age, SES, stress, SOC, and health (MWB, depression, SRH, SHC) was tested using Pearson’s product-moment correlation. Multiple linear regression analysis was applied to investigate associations between sex, age, SES, stress, SOC, and the outcome of each of SRH, SHC, MWB, and depressive symptoms. The interaction effects including combinations of sex, stress, and SOC were also tested. An assumption for conducting linear regression analysis is to have continuous variables. As stated by Wu and Leung [53], Likert scales are often treated as interval scales when included in regression analyses, when strictly speaking, it is an ordinal scale. Meanwhile, a study by Tacoby [54] also showed that the decisions used in measurement levels depended on the researcher’s interpretation of the differences among the observational categories into which the empirical objects are divided. When considering the dependent and independent variables of stress and SOC for use in the present study, the assumption of continuous variables was met as the variables were constructed as sum scores. The SES variables including mother’s and father’s education level and employment status were originally scaled at the ordinal level. In the analyses, the variables were therefore constructed as summed scores representing parents’ education and parents’ employment status. In the survey, the values ‘I don’t know’ and ‘other’ were included in the assessment of SES variables to ensure valid responses from the participants. In the regression analyses, these values were excluded, due to the assumption of including only continuous variables. Model assumptions for linear regression analysis were tested, and no indications of multicollinearity (VIF < 0.10 and tolerance > 0.02, correlations < 0.80) were found. The assumptions of linearity, homoscedasticity, and independent residuals were also met, where the Durbin Watson test were close to 2 for all models and the residuals were normally distributed through an inspection of the scatterplot [52]. The independent variables were included in the following order: (1) sex and age; (2) SES, (3) stress; (4) SOC; (5) sex × stress, and sex × SOC, and SOC × stress. The last step of the four regression models is presented in the results section; statistical significance was set to *p* ≤ 0.05.

## 3. Results

### 3.1. Mean Scores and Correlations of the Included Scales

The distribution of sex, age, and socio-economic status (SES) is presented in Table 1. When looking at sex, 580 (47%) were girls and 644 (52.2%) were boys; 9 did not report sex. Mean age was 16.62 years (SD = 1.61 years) for the total sample; for boys it was 16.68 years (SD = 1.60 years), and for girls it was 16.55 years (SD = 1.61 years). Table 2 presents an overview of the sex mean differences on the included scales. Boys scored significantly higher on SOC, MWB, and SRH, whereas girls scored significantly higher on SHC and depressive symptoms, showing weak-to-moderate strong mean differences. The correlation analysis is displayed in Table 3. The main variables of MWB, depressive symptoms, SHC, SRH, stress, and SOC showed moderate-to-strong correlations in expected directions; the strongest correlations were between SOC, depression, and MWB. The SES variables moreover showed weak to moderate strong correlations with the other variables. 

### 3.2. Regression Analyses for Variables Associated with Mental Wellbeing (MWB) and Depressive Symptoms 

Table 4 presents the results of the multiple linear regression analyses investigating the associations between sex, age, SES, stress, SOC, and the dependent variables depressive symptoms and MWB. When looking at the two models, sex was significantly related with depressive symptoms, where girls reported higher scores than boys; no significant sex differences were found on MWB. Age showed a non-significant association with MWB and a weak positive and significant association with depressive symptoms, indicating that adolescents seem to have a stable level of MWB and a weak increase in symptoms of depression across age groups. Of the SES variables, perception of stronger family economy showed a significant positive and weak association with MWB. Parents’ employment status also showed a significant and positive association with depressive symptoms. Stress was significantly positively associated with depressive symptoms (22% explained variance), but not with MWB, after being controlled for the other variables. A strong positive relation was found between SOC and MWB (20% explained variance), whereas a significant strong and inverse relation was found between SOC and depressive symptoms (24% explained variance), controlled for the other variables. Significant interaction effects were found between sex × stress on MWB, and of sex × SOC on depressive symptoms, where the associations were strongest for girls. A significant interaction effect was also found between stress × SOC on depression, indicating that the strength of the relation between stress and depressive symptoms depended on the level of SOC. The total explained variance in the two regression models was 41% in the model with MWB and 68% in the model with depressive symptoms. 

### 3.3. Regression Analyses for Variables Associated with Self-Rated Health (SRH) and Subjective Health Complaints (SHC)

When looking at the results from the regression analyses with SRH and SHC as outcome variables (Table 5), sex was significantly associated with SHC, where girls scored higher than boys. Age showed a weak, significant inverse association with SRH, but was not significantly associated with SHC. Adolescents’ perception of stronger family economy associated significantly with higher scores on SRH; the other associations including SES were non-significant. Stress was not significantly associated with either SRH or SHC. Stronger SOC was significantly associated with higher levels of SRH and lower levels of SHC. A significant interaction effect was found between stress × SOC on SRH; the other interaction effects were non-significant. The regression models totally explained 21% of the variance in SRH and 22% of the variance in SHC.

## 4. Discussion

This study investigated the role of sex, age, SES, stress, and SOC in association with four outcome variables—subjective health complaints (SHC), self-rated health (SRH), mental wellbeing (MWB), and depressive symptoms in Norwegian adolescents. 

The sex differences found in SHC were in line with previous findings showing that girls generally report more health complaints than boys [9,10,11,12]. Sex differences in depressive symptoms are well-established in the research literature, showing that girls report higher levels of depressive symptoms than boys during the adolescent years [5,6,7,8,55]. The focus in discussions has been placed on whether the symptoms represent real changes in mental health or whether especially girls’ report of higher levels of depressive symptoms and other mental health problems partly result from gender role differences, and a shift in how symptoms are perceived and reported by informants [6]; however, this might not be regarded as a key explanatory factor. In Norway, the Norwegian public health report states that the causes of the increased report of mental health problems in adolescents are complex and might be explained by a range of psychological, biological, and psychosocial factors in the different situations that adolescents partake in, as well as broader socioeconomic and cultural influences in society [1,2,8]. This points to the fact that the causes of the increased reported rates of mental health problems needs to be further investigated. 

The results showed that higher stress level associated significantly with higher levels of depressive symptoms and with lower MWB, especially in girls. The associations between stress and each of SRH and SHC were non-significant. Although exposure to stressful events is a normal part of adolescent life, exposure to multiple independent and cumulative stressors plays a substantial role in the development of mental health problems, where girls seem to be more vulnerable to the negative health effects of stress than boys [4,56]. The perceived importance of the stressor and the individual’s evaluations of the ability to cope with the stressor, are fundamental for the impact of the stressor and for the health outcomes of stress. However, one should be aware of possible reciprocal associations; just as stress experience might lead to more mental health problems, it is equally possible that mental health problems can lead to more vulnerability to perceived situations and experiences as stressful, leading to spiraling negative effects.

The findings showed support for SOC as strongly associated with adolescents’ perception of depressive symptoms and especially MWB, and weaker associations were found with SHC and SRH. Furthermore, a significant but weak moderating role of sex on the relationship between SOC and depressive symptoms was found, showing that SOC seemed to be a relevant coping resource especially for girls’ experience of depressive symptoms. When considering the interaction effects of stress by SOC, the results mainly supported a compensatory role of SOC in relation to MWB and SHC, whereas weak but significant support for a protective/buffering role of SOC was found in relation to depressive symptoms and SRH. The results thus indicated that SOC seemed to be a stronger coping resource for adolescents’ mental health, compared with SHC and SRH, despite experience of stressors [24,26,27]. Antonovsky assumed that the individual is constantly exposed to stressors in daily life that might reduce health temporarily, but in the long term, this also has the potential to strengthen the individual and help cope with stress. Through the identification and use of different resistance resources, the individual develops a strong SOC that helps one to mobilize resources to cope with stressors and manage tension successfully, which promotes movement on the positive end of the ease/dis-ease continuum [22,23,24]. 

Although no causal conclusions could be drawn, the results provide insight into the importance of stress experience and SOC, especially in association with adolescents’ report of mental health, controlled for sex, age, and SES. The findings thus support the importance of strengthening SOC in adolescents, among an array of other possible personal and social coping resources (e.g., self-esteem, self-efficacy, and resilience). Interestingly, the study showed a stronger association between stress and MWB and between SOC and depressive symptoms for girls, which shows that stress and SOC might affect girls’ and boys’ mental health differently, during adolescence. 

Working on promoting adolescents’ coping resources is important for strengthening their ability to cope with life stressors and natural ups and downs, which is important for their overall health and wellbeing. This requires cross-sectorial action that should be integrated in central developmental contexts where adolescents and adults meet on a regular basis (e.g., family, school, peers, and neighborhood) [24,34]. Although health is influenced by different areas of the adolescents’ lives, school is one important setting. In Norway, the new interdisciplinary theme of “public health and coping” has been implemented in both elementary and secondary school as part of the compulsory curriculum. This strategy presents an opportunity for implementing universal health promoting strategies focusing on coping with normative stressors in daily life and strengthening adolescents’ coping resources through socio-emotional learning and promotion of health literacy, which might also contribute to facilitating SOC [22,29]. 

### Strengths and Limitations

The strengths of this study were the use of validated instruments, the relatively large sample size, and high response rate. However, the cross-sectional design did not allow us to make conclusions regarding causality and it is possible that the variables might be reciprocally related. A longitudinal design would have been preferable in order to draw conclusions about the relative strength of the variables in predicting health outcomes. 

The data were based on self-reports from adolescents and should be evaluated with reference to potential self-reporting bias. Self-reporting requires that adolescents can understand and reflect around aspects related to health and illness (e.g., social desirability and over- and under-reporting). This might especially be relevant for the youngest adolescents, with reference to potential challenges regarding reflections on abstract concepts. The sample size could contribute to protection from the influences of potential bias related to sample selection and self-reports. The study was based on public lower- and upper-secondary schools in rural areas of mid-Norway; the findings might therefore not generalize to schools in urban areas and larger cities, and private schools. Regarding the recruitment of adolescents and administration of questionnaires, the teachers were strongly encouraged by the principal to administer the questionnaire to the students, however, administration was based on the teachers’ decision depending on time needed for educational activities. The study did not have any data on students who did not participate in the study or the parents’ mental health status, which was also a limitation of the present study. 

## 5. Conclusions

The present study showed that girls reported significantly higher levels of depressive symptoms and SHC than boys, after controlling for sex, age, SES, stress, and SOC. Stress associated with significantly higher levels of depressive symptoms, where the association between stress and depression was significantly stronger in girls. The results showed that SOC is a stronger coping resource in association with mental health (especially for girls) than with SHC and SRH. The findings also support a compensatory role of SOC on the association between stress and health during adolescence.

## Figures and Tables

**Table 1:** Demographic characteristics of the sample.

Variables	Total n (%)	
Gender		
Boys	580 (47.0)	
Girls	644 (52.2)	
Missing	9 (0.7)	
Age		
13–14 years	381 (30.9)	
15–16 years	453 (36.7)	
17–19 years	399 (32.3)	
Family economy		
Bad economy all the time	113 (9.2)	
More or less bad economy	243 (19.7)	
Neither had bad or good economy	264 (21.4)	
More or less good economy	327 (26.5)	
Good economy all the time	254 (20.6)	
Missing	32 (2.6)	
Parents’ education	Mother	Father
Primary and lower secondary school	37 (3.0)	69 (5.6)
Upper secondary school	283 (23.0)	366 (29.7)
University up to 4 years	303 (24.6)	197 (16.0)
University more than 4 years	221 (17.9)	161 (13.1)
Unknown	365 (29.6)	393 (31.9)
Missing	24 (1.9)	47 (3.8)
Parents’ job status	Mother	Father
Fulltime job	798 (64.7)	1018 (82.6)
Part-time job	238 (19.3)	86 (7.0)
Unemployed / on leave	47 (3.8)	28 (2.3)
Staying at home	83 (6.7)	32 (2.6)
Other	41 (3.3)	37 (3.0)
Missing	26 (2.1)	32 (2.6)
Total	1233 (100)	

**Table 2:** Sex mean differences on stress, sense of coherence, mental wellbeing, symptoms of depression, self-rated health, and subjective health complaints.

	Mental Wellbeing (n = 728)	Symptoms of Depression (n = 729)	Self-Rated Health (n = 1209)	Subjective Health Complaints (n = 759)	Sense of Coherence (n = 715)	Stress (n = 730)
	*Mean* (SD)	*Mean* (SD)	*Mean* (SD)	*Mean* (SD)	*Mean* (SD)	*Mean* (SD)
Girls	46.41 (9.75)	35.95 (12.88)	3.36 (1.27)	23.75 (7.70)	57.95 (13.26)	66.18 (15.59)
Boys	49.82 (9.48)	28.03 (10.71)	3.14 (1.44)	20.61 (8.70)	63.96 (12.51)	58.08 (14.15)
Total	48.08 (9.77)	32.06 (12.45)	3.25 (1.36)	22.16 (8.33)	60.88 (13.20)	62.29 (15.50)
Range	13–70	14–73	1–5	12–60	18–91	40–116
*t*-value	−4.76 ***	9.00 ***	2.91 **	5.34 ***	−6.20 ***	7.29 ***
Cohen’s *d*	0.35	0.67	0.16	0.38	0.47	0.54

Note. * *p* ≤ 0.05; ** *p* ≤ 0.01; *** *p* ≤ 0.001.

**Table 3:** Correlations between the study variables.

	MWB	D	SHC	SRH	S	SOC	Age	PE	PVS	FE
Mental wellbeing (MWB)	-	−0.58 **	−0.24 **	0.41 **	−0.33 **	0.61 **	−0.09 *	0.01	−0.10 **	0.24 **
Depression		-	0.44 **	−0.36 **	0.60 **	−0.75 **	0.14 **	−0.08 *	0.13 **	−0.22 **
Subjective health symptoms (SHC)			-	−0.25 **	0.33 **	−0.39 **	0.11 **	−0.08 *	0.04	−0.13 **
Self-rated health (SRH)				-	−0.19 **	0.37 **	−0.24 **	0.05	0.03	0.69 **
Stress					-	−0.51 **	0.15 **	−0.10 **	0.03	−0.10 **
Sense of Coherence (SOC)						-	−0.12 **	0.07	−0.11 **	0.25 **
Age							-	−0.25 **	−0.11 **	−0.21 **
Parents’ education (PE)								-	0.13 **	0.01
Parents’ vocational status (PVS)									-	0.00
Family economy (FE)										-

Note. * *p* ≤ 0.05; ** *p* ≤ 0.01.

**Table 4:** Summary of the hierarchical regression analysis for variables associated with mental wellbeing and depressive symptoms.

	Mental Wellbeing (n = 494)					Symptoms of Depression (n = 494)				
	B	*SE B*	β	95% CI	F	B	*SE B*	β	95% CI	F
Constant	47.88	4.37			34.54 ***	34.76	4.31			103.45 ***
Sex	0.36	0.72	0.02	−1.04–1.77		−3.13	0.71	−0.12 ***	−4.52–−1.75	
Age	0.23	0.20	0.04	−0.63–0.17		0.41	0.20	0.05 *	0.02–0.81	
Parents’ education	0.05	0.22	0.01	−0.37–0.48		0.27	0.21	0.03	−0.15–0.69	
Parents’ employment status	0.00	0.32	0.00	−0.62–0.63		−1.00	0.32	−0.09 **	−1.62–−0.38	
Family economy	1.08	0.42	0.10 **	0.26–1.90		−0.68	0.41	−0.05	−1.49–0.13	
Stress	−0.07	0.04	−0.11	−0.14–0.00		0.15	0.04	0.19 ***	0.08–0.22	
SOC	0.42	0.04	0.59 ***	0.34–0.50		-0.65	0.04	−0.68 ***	−0.73–−0.57	
Stress × sex	0.12	0.05	0.12 *	0.01–0.22		−0.04	0.05	−0.03	−0.14–0.07	
SOC × sex	−0.04	0.06	−0.03	−0.16–0.09		0.14	0.06	0.09 *	0.02–0.26	
Stress × SOC	0.00	0.00	0.02	−0.00–0.00		−0.01	0.00	−0.08 **	−0.01–0.00	

Note. * *p* ≤ 0.05; ** *p* ≤ 0.01; *** *p* ≤ 0.001. Sex: value 0—girls; value 1—boys. Cases deleted listwise. Adjusted R^2^ = 0.41 for model with mental wellbeing and R^2^ = 0.68 for model with depression.

**Table 5:** Summary of the hierarchical regression analysis for variables associated with self-rated health and subjective health symptoms.

	Self-Rated Health (n = 493)					Subjective Health Complaints (n = 493)				
	B	*SE B*	β	95% CI	F	B	*SE B*	β	95% CI	F
Constant	4.57	0.47			13.85 ***	26.53	4.24			14.60 ***
Sex	0.02	0.08	0.01	−0.13–0.17		−2.07	0.70	−0.12 **	−3.44–−0.71	
Age	−0.08	0.02	−0.14 **	−0.12–−0.03		0.12	0.20	0.03	−0.27–0.51	
Parents’ education	0.02	0.02	0.04	−0.02–0.07		−0.15	0.21	−0.03	−0.56–0.26	
Parents’ vocational status	0.02	0.03	0.02	−0.05–0.08		−0.55	0.31	−0.08	−1.16–0.06	
Family economy	0.14	0.05	0.13 **	0.05–0.22		−0.13	0.41	−0.01	−0.93–0.67	
Stress	0.00	0.00	0.03	−0.01–0.01		0.05	0.03	0.09	−0.02–0.12	
SOC	0.03	0.00	0.37 ***	0.02–0.03		−0.19	0.04	−0.31 ***	−0.27–−0.11	
Stress × sex	0.01	0.01	0.05	−0.01–0.02		0.05	0.05	0.06	−0.05–0.16	
SOC × sex	−0.00	0.01	−0.02	−0.02–0.01		0.01	0.06	0.01	−0.11–0.13	
Stress × SOC	0.00	0.00	0.14 **	0.00–0.00		−0.00	0.00	−0.05	−0.01–0.00	

Note. * *p* ≤ 0.05; ** *p* ≤ 0.01; *** *p* ≤ 0.001. Sex: value 0—girls; value 1—boys. Cases deleted listwise. Adjusted R^2^ = 0.21 for model with self-rated health and R^2^ = 0.22 for model with subjective health complaints.
